# Home-Based Exercise Program With Telephone Coaching: A Feasibility Study

**DOI:** 10.1093/geroni/igaa057.1296

**Published:** 2020-12-16

**Authors:** Emma Gulley, Joe Verghese, Emmeline Ayers, Tanya Verghese, Anne Felicia Ambrose

**Affiliations:** 1 Albert Einstein College of Medicine, Bronx, New York, United States; 2 Montefiore, Bronx, New York, United States

## Abstract

Exercise is crucial to maintain mobility, reduce falls and delay functional decline in older adults, but effective implementation strategies are lacking. Self-directed home-based exercise therapy is recommended by clinicians to overcome barriers such as cost, travel and availability. However, non-adherence is a major challenge due to lack of motivation, real time feedback or social support. To overcome these barriers, we conducted a feasibility study to evaluate a home-based exercise program with telephone coaching to improve mobility in frail older adults. Four non-demented, frail community-dwelling older adults were taught one of two exercise routines at our research center. The first involved complex exercises with internal and external cueing techniques that have been associated with neuroplasticity in previous studies (N=3). The second was a lesser cognitively demanding control program that included aerobic, balance and strengthening exercises (N=1). One week later, the participants were asked to repeat the exercises in their own home. The research assistant coached the patient over the telephone. A board-certified physiatrist was present during the home session to monitor adherence and fidelity to the protocol as well as address safety. The study produced qualitative findings regarding recruitment strategies, exercise feasibility, and other logistical issues relating to participant understanding, safety, and monitoring. Based on direct observation of participants at home, safety assessment protocols, instructions, and exercises were all refined. Building on this data, we plan to design a clinical trial to evaluate the impact of complex exercises designed to promote neuroplasticity and reduce cognitive and motoric decline in older adults.

